# Melatonin Promotes BMSCs Osteoblastic Differentiation and Relieves Inflammation by Suppressing the NF-*κ*B Pathways

**DOI:** 10.1155/2023/7638842

**Published:** 2023-05-24

**Authors:** Yiqiang Hu, Yuan Xiong, Kangkang Zha, Rangyang Tao, Lang Chen, Hang Xue, Chenchen Yan, Ze Lin, Yori Endo, Faqi Cao, Wu Zhou, Guohui Liu

**Affiliations:** ^1^Department of Orthopedics, Union Hospital, Tongji Medical College, Huazhong University of Science and Technology, Wuhan 430022, China; ^2^Hubei Province Key Laboratory of Oral and Maxillofacial Development and Regeneration, Wuhan 430022, China; ^3^Division of Plastic Surgery, Brigham and Women's Hospital, Harvard Medical School, Boston, MA 02152, USA

## Abstract

Bone mesenchymal stem cells (BMSCs) play an important role in maintaining the dynamic balance of bone metabolism. Recent studies have reported that a decrease in the osteogenic function of MSCs is strongly associated with osteoporosis. Melatonin is a neuroendocrine hormone produced in the pineal gland and is essential in the physiological regulation. This study is aimed at exploring the effect of melatonin on MSCs osteoblastic differentiation and elucidate the underlying mechanisms. We isolated BMSCs from rat bone marrow and demonstrated that melatonin improved osteogenic differentiation of BMSCs by the alizarin red staining and ALP staining. We then showed that melatonin enhanced osteogenic gene expression in BMSCs, including ALP, Col 1, OCN, OPN, and RUNX2. We further revealed that melatonin inhibited the inflammatory response of BMSCs by suppressing the NF-*κ*B signaling pathways. In light of this, we found that the NF-*κ*B pathway-specific activator TNF-*α* activated the NF-*κ*B pathway, inhibited osteogenic differentiation, and induced inflammatory response in BMSCs. Melatonin was found to reverse the inhibitory effect of TNF-*α* on osteogenic differentiation and inflammation in BMSCs. Taken together, these findings indicated that melatonin may have therapeutic potential to be used for the treatment of osteoporosis.

## 1. Introduction

Osteoporosis is a highly prevalent systemic bone disease characterized by bone microstructure deterioration and reduced bone mineral density, leading to bone fragility and an increased risk of fracture. Osteoporosis often happens in women due to the hormonal changes experienced by postmenopausal women, which can lead to severe bone loss [1, 2]. It is also generally regarded as a major public health problem, as it is a significant cause of disability, morbidity, and healthcare burden. Recent studies have reported that osteoporosis is associated with an imbalance between bone formation and bone resorption [3]. Bone mesenchymal stem cells (BMSCs) play an important role in maintaining the dynamic balance of bone metabolism [4]. Under pathological condition, preferential differentiation of BMSCs towards adipocytes is observed, leading to increased bone marrow fat deposition and bone loss [5]. It follows, therefore, that enhancing the osteogenesis differentiation of BMSCs may be an effective strategy for restoring bone mass and treating osteoporosis [6].

Melatonin is a hormone containing indoleamine and is mainly secreted by the pineal gland in mammals. Many studies have shown that melatonin regulates a variety of important physiological functions, such as circadian rhythms, reproduction, and neuroendocrine activity [7]. In addition, melatonin is known to prevent synovium MSCs from proinflammatory cytokines by decreasing the production of reactive oxygen species and enhancing the production of superoxide dismutase [8]. Accumulating evidence now suggests that melatonin is also associated with metabolic homeostasis of the bones. Studies have shown that melatonin inhibits osteoclast differentiation and estrogen deficiency-induced osteoporosis [9]. Furthermore, melatonin has been shown to increase the number of bone trabeculae, improve the microarchitecture of the femur and vertebrae, and enhance bone mineral density in retinoic acid–induced osteoporosis [10]. However, the effect of melatonin on BMSCs have not been completely elucidated. The potential mechanisms of melatonin on TNF-*α* induced BMSCs osteoblastic differentiation has yet to be elucidated.

NF-*κ*B, an important transcription factors, has been extensively studied in bone remodeling [11, 12]. NF-*κ*B was also considered to be a key transcription factor that regulates inflammatory responses and osteogenesis in MSCs. NF-*κ*B has ideal potential in treating inflammation-related bone remodeling [11, 13]. Recent studies have reported that activation of the NF-*κ*B pathway could inhibit bone formation and osteogenic differentiation. In the pathogenesis of osteoporosis, proinflammatory cytokines and estrogen deficiency may suppress osteoblasts by activating NF-*κ*B activity [14, 15]. Wang et al. reported that taxifolin enhances osteogenic differentiation of BMSCs via suppression of the NF-*κ*B signaling pathway [16]. However, the role of the NF-*κ*B pathway in melatonin regulating BMSCs osteoblastic differentiation has not been fully elucidated.

In this research, we will explore the effect of melatonin on BMSCs osteoblastic differentiation and inflammation. We will further investigate the role of the NF-*κ*B pathway in melatonin's effect on enhancing osteoblastic differentiation of BMSCs. We will then assess the effects of melatonin on TNF-*α*-induced BMSCs osteoblastic differentiation and inflammation.

## 2. Materials and Methods

### 2.1. Isolation and Culture of BMSCs

All experiments were approved by the Institutional Animal Care and Use Committee Tongji Medical College, Huazhong University of Science and Technology, China. As wepreviously described, bone marrow mesenchymal stem cells were isolated from 5-week-old male Sprague Dawley rats (SD rats) [17, 18]. Briefly, the tibia and femurs of SD rats were collected. Then, the tibia and femurs were sterilized by ethyl alcohol. The bone marrow was washed repeatedly with DMEM/F-12 (Gibco, USA) using a sterile 10 mL syringe. The marrow cells were then cultured in MSCs complete medium (Cyagen, USA). After 24 h, the complete medium was changed to remove nonadherent cells. Then, the medium was changed every 3 days, and the cells were observed regularly under an inverted microscope. The monolayer with 80%-90% confluence was harvested with 0.25% EDTA-trypsin and cultured in an appropriate culture plate. The cells received different concentrations of melatonin (50, 100 *μ*M) or TNF-*α* (10 ng/mL) treatment and were used for subsequent experiments.

### 2.2. Flow Cytometry

Flow cytometry was used to detect the surface markers of BMSCs as we previously described [19, 20]. In brief, BMSCs were washed with phosphate-buffered saline (PBS) and collected by trypsin. The cells were centrifuged at 1500 rpm twice. The cells were resuspended and incubated with a solution with antibodies against CD73, CD90, CD34, and CD45 for 30 min. After being incubated with the antibodies, the cells were centrifuged at 1500 rpm for 5 min. The labeled cells were washed by PBS for three times. Then, the labeled cells were resuspended in 200 *μ*L PBS and detected by flow cytometry (Becton Dickinson, USA).

### 2.3. Alizarin Red Staining

Alizarin red staining was utilized to evaluate osteogenic differentiation (Cyagen, USA) according to the protocol of the manufacturer. Briefly, BMSCs were cultured in osteogenic differentiation media in six-well plates to induce osteogenic differentiation. 21 days after the induction of differentiation, the cells were washed twice with PBS. Then, the cells were fixed in 4% paraformaldehyde for 30 min at room temperature. After being washed with PBS, the cells were stained with alizarin red staining solution for 30 min at room temperature. The cells were then rinsed with PBS three times with shaking. The results were observed by a light microscope (Olympus, Japan).

### 2.4. ALP Staining

ALP staining (Alkaline Phosphatase staining) was assessed by the BCIP/NBT alkaline phosphatase color development kit (Beyotime, China) according to the protocol of the manufacturer. In brief, BMSCs were induced to undergo osteogenic differentiation in six-well plates in osteogenic differentiation media. After the induction of osteogenic differentiation, the cells were washed twice with PBS. Then, the cells were fixed in 4% paraformaldehyde for 30 min at room temperature. The BCIP/NBT staining solution was added to the cells in the dark at room temperature. BCIP/NBT staining solution was then removed. The deionized water was used to terminate a color development reaction. The results were observed by a light microscope (Olympus, Japan).

### 2.5. RT-PCT

RT-PCR was used to assess the osteogenic mRNA genes of alkaline phosphatase (ALP), collagen type I A1 (Col 1), osteocalcin (OCN), osteopontin (OPN), runt-related transcription factor 2 (RUNX2), interleukin-1*β* (IL-1*β*), interleukin-6 (IL-6), and tumor necrosis factor-*α* (TNF-*α*). For RT-PCR, the total RNA of BMSCs was extracted by Trizol reagent (Life Technology, USA), and the cDNA was prepared from RNA using reverse transcriptase (Takara, Japan). Then the PCR was analyzed with SYBR Green Master Mix by using a real-time PCR system (Applied Biosystems, USA). Primers were designed for each target gene, as detailed in Table 1. Relative transcript expression was calculated using the 2^-*ΔΔ*Ct^ method after normalization against GAPDH. Expression data were presented as a fold increase relative to the endogenous control.

### 2.6. Western Blotting

BMSCs were seeded into six-well plates and cultured overnight. After melatonin treatment, total protein was extracted using a RIPA lysis buffer with a proteinase inhibitor cocktail. Protein was analyzed on 10–12% SDS-PAGE and transferred onto polyvinylidene fluoride (PVDF) membranes by electrophoresis. After blocking with nonfat milk for 2 h, the PVDF membranes were incubated with anti-RUNX2 (Abcam, UK), anti-OCN (Abcam, UK), anti-p-p65 (Abcam, UK), anti-p65 (Abcam, UK), anti-IkB*α* (Abcam, UK), anti-p-IkB*α* (Abcam, UK), and anti-GAPDH (Abcam, UK) primary antibodies at 4°C overnight. Next, the membranes were washed for 10 minutes with TBS-T three times and incubated with appropriate secondary antibodies for 1 h at room temperature. After three washes with TBS-T, the membranes are visualized using the enhanced chemiluminescence method according to the manufacturer's instructions.

### 2.7. Statistical Analysis

All experiments were conducted at least three times and presented as the mean ± standard deviation. One-way ANOVA, followed by Tukey's multiple comparison test was used to analyze differences in more than two groups by using GraphPad Prism (GraphPad Software). *P* < 0.05 was considered statistically significant.

## 3. Results

### 3.1. Characterization of BMSCs

**Figure 1:**
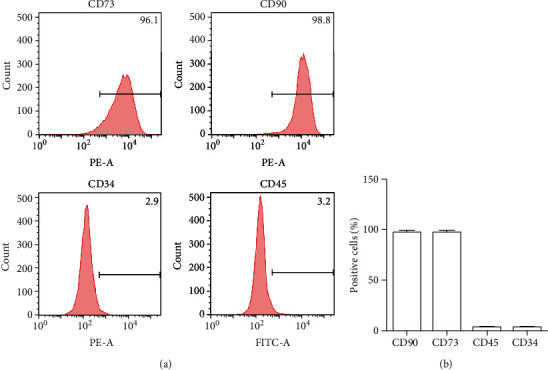
Identification of BMSCs. (a) Flow cytometry was utilized to assess the expressions of CD34, CD45 CD73, and CD90 surface markers on the BMSCs. (b) Quantification analysis of positive cells in BMSCs.

In this study, we isolated and cultured BMSCs from rat bone marrow. We used flow cytometry to detect surface markers of BMSCs. The results demonstrated that the cells isolated from rat bone marrow highly expressed specific surface markers of stem cells, such as CD73 and CD90. The positive rates of these cells were greater than 95%. We also found that the cells lowly expressed CD34 and CD45 (Figures 1(a) and 1(b)). These results indicated that we isolated BMSCs from rat bone marrow successfully.

### 3.2. Melatonin Promotes Osteogenic Differentiation of BMSCs

**Figure 2:**
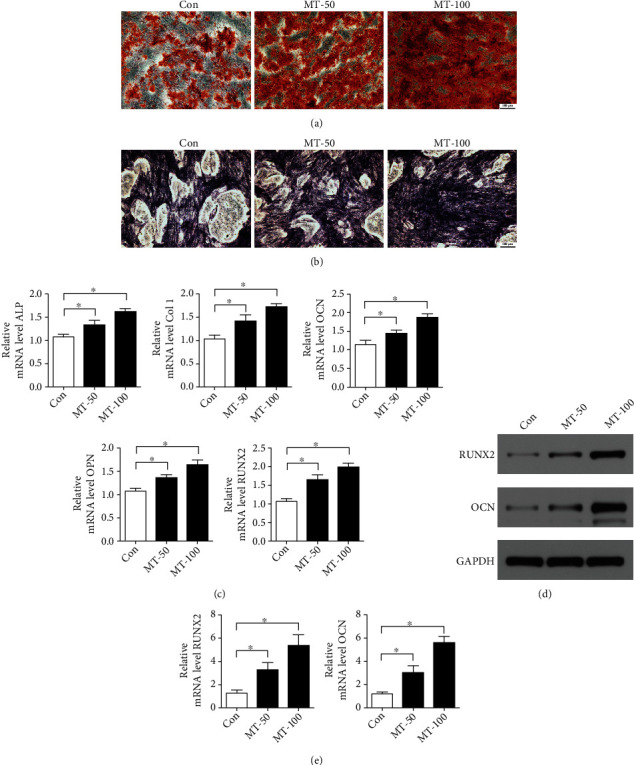
Melatonin promotes osteogenic differentiation of BMSCs. (a) Calcium deposits were visualized by alizarin red staining. Scale bar: 100 *μ*m. (b) ALP staining was used to evaluate ALP activity. Scale bar: 100 *μ*m. (c) RT-PCR was used to quantify the expression of osteogenic genes in BMSCs, such as ALP, Col 1, OCN, OPN, and RUNX2. (d, e) Western blotting was used to investigate the protein expressions of RUNX2 and OCN. MT-50: 50 *μ*M melatonin; MT-100: 100 *μ*M melatonin. The data are expressed as the mean ± SD from three independent experiments. ^∗^*P* < 0.05 versus the control (Con) group.

To evaluate the effect of melatonin on osteogenic differentiation of BMSCs, we used alizarin red staining. We found that greater calcium deposits were formed in the melatonin group than in the control group. The effect of melatonin on enhancing calcium deposition was greatest at a dose of 100 *μ*M (Figure 2(a)). To further investigate the effect of melatonin on the osteogenic ability of BMSCs, ALP staining was used. The results revealed that melatonin enhanced ALP activity in the BMSCs compared with the control group (Figure 2(b)). In addition, RT-PCR was performed to detect osteogenic gene expression in BMSCs. The results of RT-PCR illustrated that the melatonin promoted osteogenic gene expression by BMSCs cells, such as ALP, Col 1, OCN, OPN, and RUNX2, and that the mRNA levels of osteogenic genes in the 100 *μ*M melatonin group were markedly higher compared with those of the 50 *μ*M melatonin group (Figure 2(c)). The results of western blotting showed that melatonin significantly increased the protein levels of RUNX2 and OCN (Figures 2(d) and 2(e)). Collectively, these results showed that melatonin treatment enhanced osteogenic differentiation of BMSCs.

### 3.3. Melatonin Inhibited Inflammatory Response in BMSCs

**Figure 3:**
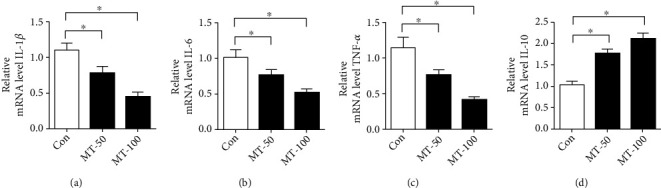
Melatonin inhibited inflammatory response in BMSCs. (a–c) RT-PCR was used to detect the expression of proinflammatory genes, such as IL-1*β*, IL-6, and TNF-*α* in BMSCs. (d) RT-PCR was used to detect anti-inflammatory IL-10 gene expression in BMSCs. MT-50: 50 *μ*M melatonin; MT-100: 100 *μ*M melatonin. Data are expressed as the mean ± SD from three independent experiments. ^∗^*P* < 0.05 versus control (Con) group.

To elucidate the potential mechanisms underlying the effects of melatonin on BMSC osteogenic differentiation, we used RT-PCR to quantify inflammatory gene expression in BMSCs. The results demonstrated that melatonin significantly decreased the level of proinflammatory gene expression, including IL-1*β*, IL-6, and TNF-*α* in BMSCs. The inhibitory effect of melatonin was greatest at 100 *μ*M compared with the 50 *μ*M melatonin group (Figures 3(a)–3(c)). In addition, we found that melatonin significantly increased the mRNA level of the anti-inflammatory gene IL-10 in BMSCs compared to the control group. Similarly, the effect of melatonin on IL10 mRNA level was significantly greater in the 100 *μ*M melatonin group compared with the 50 *μ*M melatonin group (Figure 3(d)). Those results demonstrated that melatonin inhibited the inflammatory response in the BMSC.

### 3.4. Melatonin Suppressed TNF-*α*-Caused Activation of the NF-*κ*B Signaling Pathways in BMSCs

**Figure 4:**
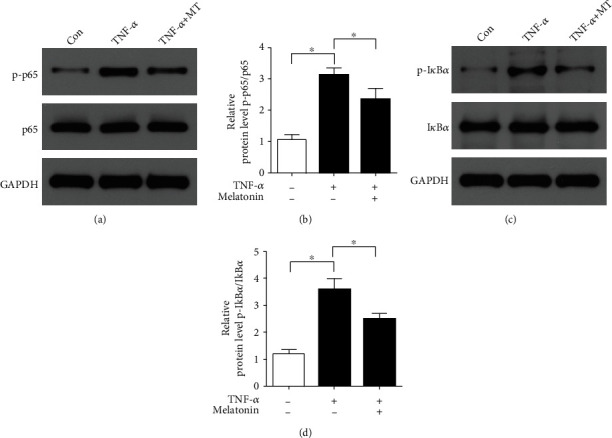
Melatonin suppressed TNF-*α*-induced NF-*κ*B signaling pathway activation in BMSCs. (a) Western blotting was used to investigate NF-*κ*B pathway-related proteins such as p65 and p-p65. (b) Quantitative analysis of the protein levels of p-p65/p65. (c) Western blotting was used to investigate NF-*κ*B pathway-related proteins such as IkB*α* and p-IkB*α*. (d) Quantitative analysis of the protein levels of p-IkB*α*/IkB*α*. Melatonin: 100 *μ*M; TNF-*α*: 10 ng/mL. The data are expressed as the mean ± SD from three independent experiments. ^∗^*P* < 0.05 versus the control (Con) group.

To investigate the effects of melatonin on the NF-*κ*B pathway, the NF-*κ*B pathway-specific activator TNF-*α* was used to activate NF-*κ*B pathway in BMSCs. Western blotting was utilized to detect the expression of NF-*κ*B pathway-related proteins. The results of western blotting showed that TNF-*α* significantly increased the levels of p-p65 and p-IkB*α* protein expression, which indicated that TNF-*α* activated the NF-*κ*B pathway. Melatonin treatment significantly decreased the levels of p-p65 and p-IkB*α* protein expression induced by TNF-*α*. The inhibitory effect of melatonin on protein levels was greatest at the dose of 100 *μ*M compared with the 50 *μ*M melatonin group (Figures 4(a)–4(d)). These results illustrated that melatonin suppressed the NF-*κ*B signaling pathway activation by TNF-*α* in BMSCs.

### 3.5. Melatonin Reverses the Inhibitory Effect of TNF-*α* on Osteogenic Differentiation in BMSCs

**Figure 5:**
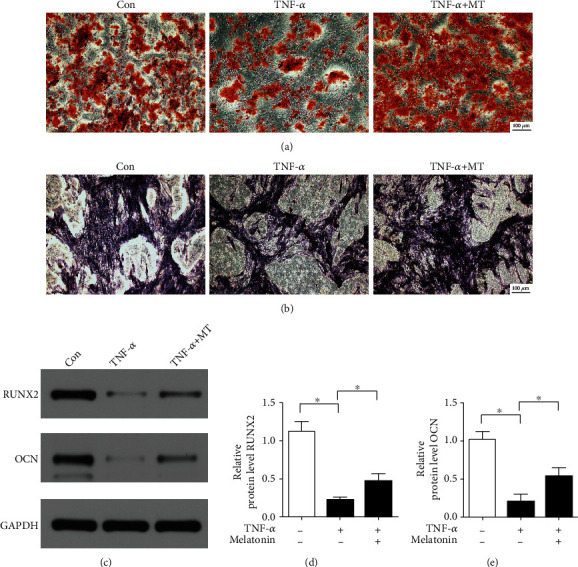
Melatonin reverses the inhibitory effect of TNF-*α* on osteogenic differentiation in BMSCs. (a) Calcium deposits were observed by alizarin red staining. Scale bar: 100 *μ*m. (b) ALP staining was used to evaluate the ALP activity. Scale bar: 100 *μ*m. (c-e) Western blotting was used to investigate the protein expression of RUNX2 and OCN. Melatonin: 100 *μ*M; TNF-*α*: 10 ng/mL. The data are expressed as the mean ± SD from three independent experiments. ^∗^*P* < 0.05 versus the control (Con) group.

To study the effect of melatonin on osteogenic differentiation induced by TNF-*α* in BMSCs, alizarin red staining was used to evaluate osteoinductivity. The results demonstrated that TNF-*α* inhibited calcium deposition compared with the control group in BMSCs, and melatonin alleviated the inhibitory effect of TNF-*α* on calcium deposit formation by BMSCs (Figure 5(a)). In addition, we used ALP staining to investigate the effect of melatonin on osteogenic differentiation induced by TNF-*α*. The results of ALP staining revealed that TNF-*α* suppressed the ALP activity in BMSCs, while melatonin relieved the inhibitory effect of TNF-*α* on ALP activity in BMSCs (Figure 5(b)). Next, we used western blotting to detect osteogenic-related protein expression to further study the effect of melatonin on osteogenic ability induced by TNF-*α*. The results showed TNF-*α* decreased the expression of osteogenic-related expression proteins such as RUNX2 and OCN, while melatonin significantly alleviated the inhibitory effect of TNF-*α* on RUNX2 and OCN protein expression (Figures 5(c)–5(e)). These results suggest that melatonin reversed the inhibitory effect of TNF-*α* on osteogenic differentiation in BMSCs.

### 3.6. Melatonin Relieve the Inhibitory Effect of Osteogenic- and Inflammation-Related Gene Expression Induced by TNF-*α*

**Figure 6:**
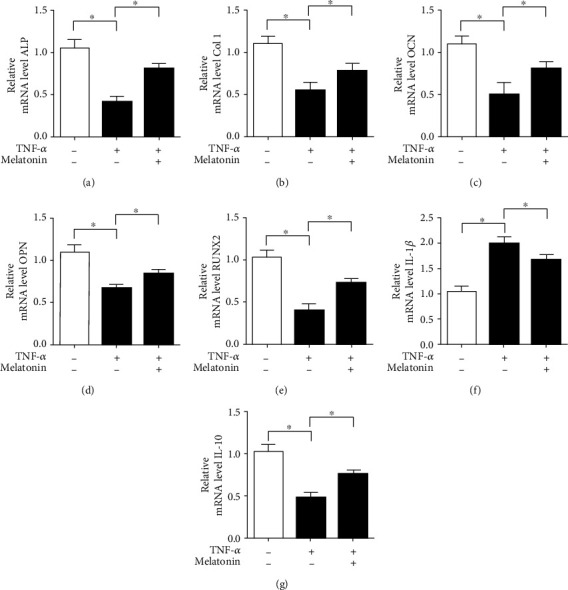
Melatonin relieves the inhibitory effect of TNF-*α* on osteogenic and inflammatory gene expression. (a–e) RT-PCR was used to detect the expression of osteogenic genes such as ALP, Col 1, OCN, OPN, and RUNX2 in BMSCs. (f) RT-PCR was used to detect the proinflammatory IL-1*β* gene expression in BMSCs. (g) RT-PCR was used to detect the anti-inflammatory IL-10 gene expression in BMSCs. Melatonin: 100 *μ*M; TNF-*α*: 10 ng/mL. Data are expressed as the mean ± SD from three independent experiments. ^∗^*P* < 0.05 versus the control (Con) group.

We used RT-PCR to detect osteogenic gene expression to further characterize the effect of melatonin on osteogenic ability induced by TNF-*α*. The results revealed that TNF-*α* decreased the mRNA levels of osteogenic genes such as ALP, Col 1, OCN, OPN, and RUNX2 compared with the control group. And melatonin promoted the mRNA expression levels of osteogenic genes including ALP, Col 1, OCN, OPN, and RUNX2 compared with the TNF-*α* group (Figures 6(a)–6(e)). We further evaluated the inflammatory gene expression by RT-PCR. The results of RT-PCR illustrated that TNF-*α* elevated the mRNA level of the proinflammatory IL-1*β* gene, while melatonin treatment resulted in a lower IL-1*β* mRNA level compared with the control group (Figure 6(f)). In addition, we found that TNF-*α* treatment decreased the anti-inflammatory IL-10 gene level compared with the control group. In contrast, melatonin alleviated the inhibitory effect of TNF-*α* on the IL-10 gene expression level (Figure 6(g)). These results indicated that melatonin relieved the inhibitory effect of TNF-*α* on osteogenic and inflammatory gene expression.

## 4. Discussion

In this study, we successfully isolated BMSCs from rat bone marrow and found that melatonin treatment enhanced osteogenic differentiation of BMSCs. Our study revealed that melatonin inhibited the inflammatory response in BMSCs by suppressing the NF-*κ*B signaling pathways. Melatonin was found to further reverse the inhibitory effect of TNF-*α* on osteogenic differentiation and proinflammatory effects in BMSCs.

Osteoporosis is a systemic metabolic disease characterized by low bone mass, altered bone microstructure, increased bone fragility, and an increased risk of fracture. Osteoporosis is caused by a disturbance in the balance of osteoclast and osteoblast functions, resulting in a negative bone remodeling balance where bone resorption surpasses bone formation [21, 22]. BMSCs are important cells in bone formation and bone reconstruction and can differentiate into osteoblasts and form bone cells [23, 24]. Recent studies have reported that the decrease in the osteogenic functions of MSCs is strongly associated with osteoporosis. Shen et al. reported that fat mass and obesity-associated protein inhibited osteoblast differentiation of BMSCs and promoted the shift of BMSCs' fate towards adipocyte, and this led to a bone formation disorder and the development of osteoporosis [25]. The results of these studies indicate that an effective means of promoting osteogenic differentiation of BMSCs may offer a therapeutic target to treat osteoporosis.

Melatonin is a neuroendocrine hormone produced in the pineal gland and is essential in the physiological regulation. A recent study reported that melatonin increased the nucleus pulposus cell viability and inhibited cell apoptosis, providing proof-of-concept for melatonin-based therapy for intervertebral disc degeneration [26]. Qiu et al. reported that melatonin increased cell viability and protected cells against pyroptosis in human stem cell-derived cardiomyocytes. Their results indicated that melatonin exerted an antipyroptotic function in cardiomyocytes and was considered as a promising agent for treating septic heart injury [27]. Interestingly, the evidence from prior studies showed that melatonin can suppress the osteoblasts ferroptosis through activating the Nrf2/HO-1 signaling pathway to improve bone microstructure, which in turn indicated that melatonin may be effective for the treatment of diabetic osteoporosis [28]. In this study, we showed that melatonin treatment enhanced osteogenic differentiation of BMSCs and inhibited the inflammatory response in BMSCs.

NF-*κ*B serves as an important transcription factor in immune cells and regulates gene expression of antiapoptotic factors, adhesion molecules, cytokines, and the overall inflammatory response. The classical NF-*κ*B pathway is regulated by the IKK*β*, which is activated by the inflammatory cytokines, such as TNF-*α* [29, 30]. Recent studies have reported that activation of the NF-*κ*B pathway is closely related to osteoporosis. Chen et al. reported that plastrum testudinis extract inhibited osteoclastogenesis and relieved bone loss by regulating the NF-*κ*B signaling pathway, demonstrating its therapeutic value in treating senile osteoporosis [31]. In addition, other studies have reported that cadmium induces cellular senescence of BMSCs through the NF-*κ*B signaling pathway and contributes to early osteoporosis development and failure in bone defect repair [32]. Zhu et al. showed that glaucocalyxin A relieved RANKL-induced osteoclastogenesis and bone resorption of bone by inhibiting the NF-*κ*B pathway activation induced by RANKL [33]. The results of this study demonstrated that melatonin suppressed TNF-*α*-induced activation of the NF-*κ*B signaling pathways and alleviated the inhibitory effect of TNF-*α* on osteogenic differentiation and inflammatory response in BMSCs.

However, there were some limitations to this research. Firstly, the study was conducted in vitro, and the findings are not necessarily indicative of what happens in vivo. Besides, it was difficult to acquire healthy human BMSCs. Therefore, we used BMSCs from SD rats to investigate the effect of melatonin on BMSCs' osteoblastic differentiation. If possible, it will be necessary to study the effect of melatonin on human BMSCs in the future.

## 5. Conclusions

In this study, we found that melatonin enhanced osteogenic differentiation of BMSCs. Our research revealed that melatonin inhibited the inflammatory response of BMSCs by regulating the NF-*κ*B signaling pathways. Furthermore, melatonin alleviated the inhibitory effect of TNF-*α* on osteogenic differentiation and the proinflammatory effect in BMSCs. Taken together, these findings indicate that melatonin has therapeutic potential in osteoporosis treatment.

## Figures and Tables

**Table 1:** Primer sequence.

Gene	Primer	Sequence
ALP	Forward	GGCGTCCATGAGCAGAACTACATC
	Reverse	CAGGCACAGTGGTCAAGGTTGG
COL 1	Forward	TGTTGGTCCTGCTGGCAAGAATG
	Reverse	GTCACCTTGTTCGCCTGTCTCAC
OCN	Forward	GGACCCTCTCTCTGCTCACTCTG
	Reverse	ACCTTACTGCCCTCCTGCTTGG
OPN	Forward	GACGATGATGACGACGACGATGAC
	Reverse	GTGTGCTGGCAGTGAAGGACTC
Runx2	Forward	CTTCGTCAGCGTCCTATCAGTTCC
	Reverse	TCCATCAGCGTCAACACCATCATTC
IL-1*β*	Forward	AATCTCACAGCAGCATCTCGACAAG
	Reverse	TCCACGGGCAAGACATAGGTAGC
TNF-*α*	Forward	ATGGGCTCCCTCTCATCAGTTCC
	Reverse	CCTCCGCTTGGTGGTTTGCTAC
IL-6	Forward	ACTTCCAGCCAGTTGCCTTCTTG
	Reverse	TGGTCTGTTGTGGGTGGTATCCTC
IL-10	Forward	GGCAGTGGAGCAGGTGAAGAATG
	Reverse	TGTCACGTAGGCTTCTATGCAGTTG
GAPDH	Forward	GACATGCCGCCTGGAGAAAC
	Reverse	AGCCCAGGATGCCCTTTAGT

## Data Availability

The data used to support the findings of this study are available from the corresponding author upon request.
